# Transchromosomic cell model of Down syndrome shows aberrant migration, adhesion and proteome response to extracellular matrix

**DOI:** 10.1186/1477-5956-7-31

**Published:** 2009-08-28

**Authors:** Frédéric Delom, Emma Burt, Alex Hoischen, Joris Veltman, Jürgen Groet, Finbarr E Cotter, Dean Nizetic

**Affiliations:** 1 Queen Mary University of London, Institute of Cell and Molecular Science, Barts and the London School of Medicine and Dentistry, 4 Newark Street, London E1 2AT, UK; 2 Radboud University Nijmegen Medical Centre, Nijmegen, the Netherlands

## Abstract

**Background:**

Down syndrome (DS), caused by trisomy of human chromosome 21 (HSA21), is the most common genetic birth defect. Congenital heart defects (CHD) are seen in 40% of DS children, and >50% of all atrioventricular canal defects in infancy are caused by trisomy 21, but the causative genes remain unknown.

**Results:**

Here we show that aberrant adhesion and proliferation of DS cells can be reproduced using a transchromosomic model of DS (mouse fibroblasts bearing supernumerary HSA21). We also demonstrate a deacrease of cell migration in transchromosomic cells independently of their adhesion properties. We show that cell-autonomous proteome response to the presence of Collagen VI in extracellular matrix is strongly affected by trisomy 21.

**Conclusion:**

This set of experiments establishes a new model system for genetic dissection of the specific HSA21 gene-overdose contributions to aberrant cell migration, adhesion, proliferation and specific proteome response to collagen VI, cellular phenotypes linked to the pathogenesis of CHD.

## Background

Down syndrome (DS), a congenital condition caused by the trisomy of human chromosome 21 (HSA21), is the most frequent chromosomal abnormality in live births associated with mental retardation and congenital heart defect (CHD) [1]. Although the pathology of DS is associated with a number of complex manifestations [1-3], the presence of a congenital heart defect (CHD) is the greatest risk factor for death during infancy. Approximately 40% of liveborn DS infants are born with a CHD [4], the majority of which involve abnormal development of the atrioventricular canal (AVC). Although CHD occur as isolated abnormalities in otherwise normal children, >50% of AVC defects are diagnosed in DS infants [5]. The association between DS and AVC defects has led to speculation that proteins encoded on chromosome 21 are involved in cardiac valve development. Molecular mechanisms responsible for the development of AVC defects are not known, and their protein cause has not yet been firmly assigned to trisomic contributions of specific HSA21 genes. A currently accepted stochastic model [6] predicts that increased cell adhesion causes the decreased migration of trisomy 21 cells preventing normal AVC formation. The crucial underlying process is the cellular response to changes in extracellular matrix, which can also trigger epithelial-mesenchymal transformation (EMT) [7], the developmental process thought to be responsible for heart valve and septa development [8]. Jongewaard *et al. *have compared integrin-mediated cell adhesive properties for skin fibroblasts isolated from DS and non-DS individuals on fibronectin (FN) and type I and VI collagen (Col I and Col VI) [9]. While cells demonstrated similar adhesion profiles to FN and Col I, all DS fibroblasts displayed an aberrantly increased adhesive capacity for Col VI compared to non-DS fibroblasts. Col VI, encoded on HSA21, is a component of the extracellular matrix that is speculated to anchor cells within the three dimensional tissue space through binding to cell surface integrins and other structural matrix components [10]. During heart development, Col VI is expressed within the endocardial cushions and the developing AVC in a pattern that parallels cell migration and septum-valve remodelling [11-13]. Though molecular screening of families with DS infants had demonstrated an association between genetic variations in the collagen VI (Col VI) region and AVC defects [14], the trisomy of the Col VI gene alone has been ruled out as the cause of CHD in DS by two reports: (i) studies of correlation of partial trisomies of HSA21 with the occurrence of CHD narrowed down a region containing some 20% of HSA21 genes, but excluding ColVI [15]; and (ii) a ColVI transgenic mouse model showed no abnormalities in heart development [16]. Therefore, other approaches are needed to examine the contribution of HSA21 proteins in the CHD-critical region to the pathogenesis of CHD.

Here we show that increased DS cell adhesion to ColVI as matrix, aberrant proliferation of adhering DS cells, and aberrant cell migration (independent of adhesion) can all be reproduced in a transchromosomic model of DS (mouse fibroblasts a bearing supernumerary human chromosome 21) [14]. Transchromosomic models of DS offer a further advantage of the possibility of specific transcriptional silencing of a single gene from the supernumerary human chromosome while maintaining the trisomic expression of all other HSA21 genes [17], thereby assigning the causative genetic contribution for a phenotype to the trisomic overdose of a single HSA21 gene [17]. We show that trisomy 21 cells, to a much greater degree than normal controls, acquire SELDI-TOF-MS detectable proteome changes specific to the presence of collagen VI as adhesion matrix. Our data provide an indication, at the proteomic level, that trisomy 21 affects the cell-autonomous proteome response to the change in the extracellular matrix composition. This set of experiments also establishes a cellular model system capable of dissecting the specific HSA21 gene-overdose contributions to aberrant cell migration, adhesion, and the proteome response to collagen VI, potentially advancing the understanding of molecular mechanisms behind the pathogenesis of CHD.

## Results

### Presence, integrity and expression of HSA21 in the transchromosomic cell WA17

**Figure 1:**
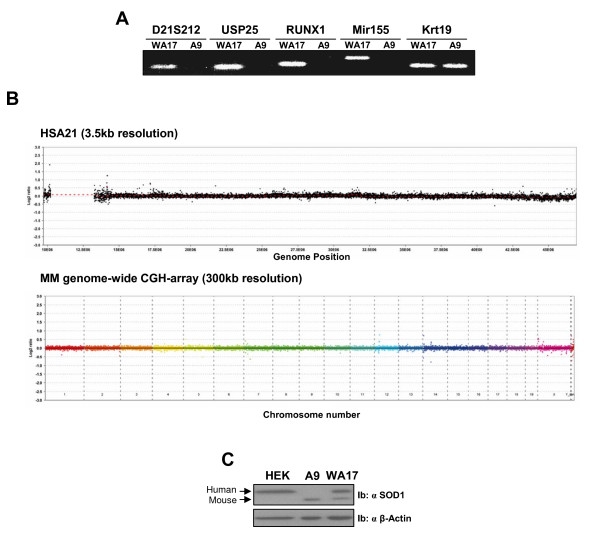
**Characterization of the integrity of the WA17 cell line model of DS**. (A) Total DNA extracted from A9 and WA17 cells was subjected to PCR analysis using human-specific primers for D21S212, USP25, RUNX1, Mir155 and mouse Krt19 primers as described in Materials and Methods. (B) Ultra high resolution array CGH hybridisation of the WA17 against A9 genomic DNA to the ultra high resolution NimbleGen HG18 chromosome 21 specific 385K arrays show the presence of the full length of HSA21 with no cryptic deletions or duplications. A 50× averaging window was also calculated, resulting in 3500 bp segments for this array. The lack of copy number rearrangements of the mouse genome between the WA17 and their parental control A9 cell lines was verified using MM8 WG CGH Whole Genome Tiling Arrays. Data were visualized in SignalMap V1.9 software (Roche NimbleGen, Wisconsin, USA). Averaging windows were used for breakpoint determination. (C) The total cell lysates of HEK 293, A9 and WA17 cells were immunoblotted with an anti-SOD1 polyclonal antibody. The mouse SOD1 (Mouse) and human SOD1 (Human) are indicated by arrows.

In this study, we used the transchromosomic mouse-human hybrid cells, WA17, which contain a supernumerary human chromosome 21 (HSA21) and are derived from a hybrid cell line obtained by fusion of mouse A9 cells with human WI-38 fibroblasts [18]. In order to confirm the presence of HSA21 in our model, we performed a PCR using human specific primer sets for markers/genes in different regions of HSA21. All four tested markers specifically amplified WA17, but not A9 DNA, establishing the presence of HSA21 (Fig 1A). Next, we examined the integrity of the genetic material in the cell lines used, to exclude any structural and copy number changes occurring during cell line establishment/propagation. The integrity of the supernumerary HSA21 in WA17 cells was examined using hybridisation to the HSA21 specific high-resolution Nimblegen arrays (HG18 CHR21 FT Chromsome 21 Tiling Array). This analysis showed (Fig 1B) that all segments of HSA21 were present in equal dose, ruling out any deletions or duplications of HSA21 down to the resolution of 3500 bp. Also, the lack of any copy number rearrangements of the mouse genome between the WA17 and their parental control A9 cell lines was verified using hybridisation to MM8 WG CGH Whole Genome Tiling Arrays, where no rearrangements were seen (Fig 1B). In addition, to confirm the expression of human proteins encoded on HSA21, the A9 and WA17 cell extracts were immunoblotted using a polyclonal a-SOD1 antibody raised against both the human and mouse SOD1 (Fig 1C). Using this antibody, we compared expression of SOD1 in the human cell line HEK 293 to WA17 and A9 cell lines. As expected, both mouse and human SOD1 proteins were detected in WA17 cell line (Fig 1C). Therefore, the "quality-control" experiments demonstrated the presence, integrity and expression of HSA21 in the transchromosomic cell WA17 (Fig 1A, B and 1C).

### Aberrant migration, adhesion and proliferation in a transchromosomic cell model of DS

**Figure 2:**
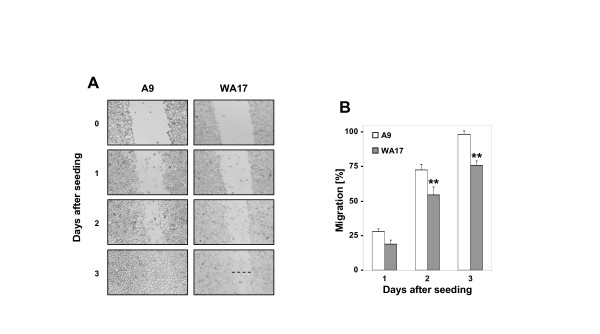
**Cell migration of the WA17 cell line model of DS**. (A) A9 and WA17 cells were grown until confluent, and treated with 10 mg/ml mitomycin C for 2 h, before a scratch wound was introduced using a yellow pipette tip. Cells were then incubated for 3 days in normal growth media. Pictures were taken at 0, 1, 2 and 3 days after wounding. (B) Pictures from 4 non-overlapping scratch wounds of each of 3 independent cultures (n = 12 data points for each cell line) from part A were taken and cells migrated into the wound site were quantified (Open bars A9, filled bars WA17). Data are presented as mean ± SD (**p < 0.001 *t*-test comparing A9 *versus *WA17).

A stochastic scenario determined by computer modelling [6] predicted that increased adhesion causes the decreased migration of trisomy 21 cells preventing normal endocardial cushion closure and AVC formation. Decreased cell migration has never been, to our knowledge, previously assayed *in vitro *in DS cells. We decided to quantitate cell adhesion to uncoated culture dish surface using the standard assay, and cell migration using the monolayer-scratch assay [19,20]. Cells were pre-treated with mitomycin-C to stop cell divisions. As can be seen in Fig 2A and 2B, the monolayer-scratch assay showed that the number of cells that migrated was significantly reduced for WA17 cells compared to control cells (A9). Quantitation of these data is shown in Fig 2B, where the percentage of migrating A9 and WA17 cells at day 1, 2 and 3 after wounding the cell monolayer is calculated. These assays indicated that defective fibroblast migration can indeed be demonstrated for DS-model cells in vitro. There was no difference in the level of cell adhesion to uncoated dish surface between the WA17 and A9 cells (data not shown). Therefore, the further novel conclusion from these data is that the decreased cell migration is a stand-alone cellular phenotype, not accompanied by increased cell adhesion to the same surface in which both assays were performed.

**Figure 3:**
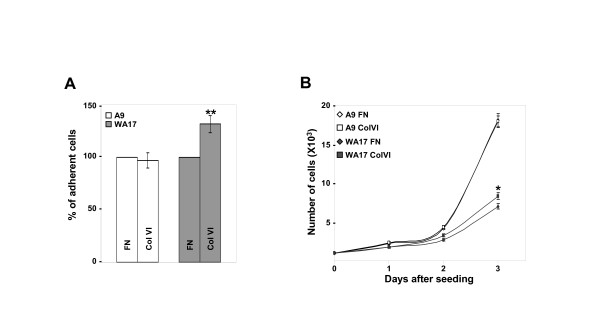
**Cell adhesion and proliferation of the WA17 cell line model of DS**. (A) Plates were coated with FN (5 μg/ml) or Col VI (10 μg/ml). A9 (open bars) and WA17 (gray filled bars) cell lines (n = 12 independent cultures for each cell line and each ECM condition) were allowed to adhere to the protein coated plates for 60 minutes. Adhesion assays were performed as described in Materials and Methods. The percentage of adherent cells are shown as mean ± SD. (**p < 0.001 *T*-test comparing FN and Col VI conditions for WA17). (B) Proliferation assay was performed as described in Materials and Methods, on plates coated with ColVI and FN, as above. Data represent the means ± SD (*p < 0.01 *T*-test comparing FN and Col VI conditions for WA17).

It has been previously demonstrated that fibroblasts explanted from the skin of DS and non-DS individuals differ in their adhesion properties to different components of the extracellular matrix (ECM). They had similar adhesion profiles to fibronectin (FN) and collagen I (Col I), but all DS cells displayed increased adhesive capacity for collagen VI (Col VI) compared to non-trisomic fibroblasts [9]. Col VI is an HSA21 gene, it is expressed in the developing AV canal extracellular matrix and it is overexpressed in DS skin tissue [21]. In order to attempt reproducing these results in the transchromosomic model of DS, A9 and WA17 cells were plated on FN or Col VI in the same conditions as previously published for fibroblasts explanted from DS donor individuals [9]. The adherence efficiency was defined by calculating the number of cells that remained attached. We confirmed that the proportion of WA17 cells adhering to ColVI was significantly increased compared the cells adhering to FN (Fig 3A). The same comparison for normal (A9) cells showed no difference (Fig 3A), fully reproducing the adhesion results from DS skin fibroblast donors [9]. Since adhesion of trisomic cells to Col VI was increased, we decided to examine the effects of the ECM type on cell proliferation. As expected, we observed a great difference in cell proliferation rates between the two cell lines, A9 and WA17 (Fig 3B). The WA17 cells had a significantly reduced proliferation rate compared to normal (A9) cells, on both adhesion matrices (FN and ColVI). This finding is in full concordance with the results obtained with cultured fibroblasts from DS donors, which had a significantly slower proliferation rate compared to fibroblasts of normal donors [22]. Interestingly, the presence of Col VI as adhesion matrix, as compared to FN, provoked a statistically significant increase in the proliferation rate of WA17, but not of A9 cells (approximately 10%).

In conclusion, we found that transchromosomic DS-model cells showed adhesion-independent defective migration on an uncoated surface, and reproduced the cellular phenotypes observed in fibroblasts from the skin of DS individuals: slower proliferation rate compared to normal cells and aberrantly increased adhesion to Col VI. In addition, trisomic cells proliferated slightly faster on Col VI than on FN, a phenotype not shared by euploid cells.

### Effect of adhesion matrix type on the proteomic profile of DS-model and normal cells

As the ECM in DS contains a higher dose of ColVI [21], it could provide signals inducing a response in cells, irrespective of the trisomy of HSA21 (the so called "outside-in" signalling perturbation [9]). On the other hand, overdose of HSA21 proteins could perturb the signalling in a cell-autonomous way, caused by the increased dose of specific unknown HSA21 proteins (the "inside-out" signalling perturbation) [9]. In order to estimate the degree to which these two conceptual mechanisms play a role in DS cells, we sought a rapid method to detect changes in the proteome composition of the cells cultured on two adhesion matrices, ColVI and FN.

**Figure 4:**
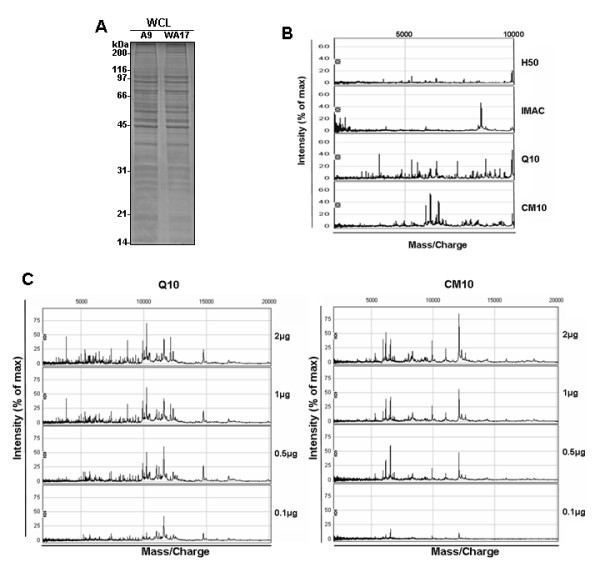
**Representative SELDI-TOF MS spectra from A9 samples**. (A) A9 and WA17 cells were lysed and proteins resolved by SDS-PAGE. Equal amounts of total protein lysate were used for electrophoresis (20 μg). (B) An example of SELDI protein-profiling spectra of 2 μg of whole cell protein extract of A9 cell line on four different chromatographic arrays (H50, IMAC, Q10 and CM10) is shown. (C) A dilution series of A9 cell protein extract sample was prepared and 2 μl of each was analyzed on a Q10 and CM10 ProteinChip array. The total amount of protein analyzed was 2, 1, 0.5 and 0.1 μg, respectively.

We first cultured WA17 and A9 cells for 3 days, resolved protein extracts by SDS-PAGE and stained with coomassie blue. A reproducible separation of the protein extracts showed SDS-PAGE patterns gave no obvious differences (Fig 4A), showing that there are no pleiotropic alterations of abundant proteins between A9 and WA17 cells.

**Figure 5:**
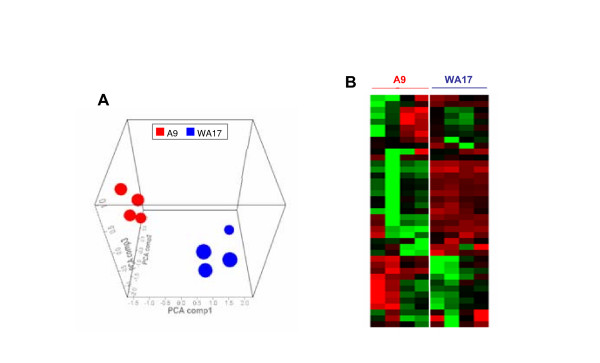
**Proteins profiles of A9 and WA17 cell lines**. (A) The intensity of peaks from SELDI spectra were analyzed using the PCA algorithm (CiphergenExpress software). A 3D PCA scores plot indicates clusters of A9 (in red) and WA17 (in blue) cell lines. (B) The heat-map view provides an at-a-glance view of spectra and their relative expression levels. Red indicates enhanced expression and green indicates reduced expression whereas black means no change in expression. The dendrogram displays clusters of spectra based on similarity of their profiling patterns. The clustering analysis indicates a separation into two main groups, namely the profiles of A9 and WA17 cell lines.

We then analysed protein extracts from each cell line (A9 and WA17) on the ProteinChip system (Ciphergen Biosystems, Fremont, CA), which is based on the integration of chemically modified array surfaces with surface-enhanced laser desorption/ionization (SELDI) time-of-flight (TOF) mass-spectrometry (MS) detection [23]. This technology was chosen as the immediate goal of our experimental approach was not the identification of biomarkers, but merely a rapid detection of a cell autonomous response to the changes in the composition of ECM, and the influence (if any) of trisomy 21 on this response. Protein profiles of the A9 cell line were measured on four different chemically modified array surfaces: H50, IMAC, Q10, and CM10 arrays by SELDI-TOF-MS. Spectra of protein extracts (2 μg protein per spot) revealed about 50-150 peaks per array type (Fig 4B). As expected, due to specific binding affinities of the chromatographic surfaces used, different portions of the A9 proteome were retained and detected on different surfaces. Highest peak numbers and densities were achieved on the arrays Q10 and CM10. In an attempt to determine the optimum amount of spotted protein for SELDI profiling, we have used a range of protein quantities (2, 1, 0.5 and 0.1 μg) per spot of an array, of A9 cell lysate (Fig 4C). From these experiments, the highest peak numbers were achieved on the array Q10 for 2 μg of protein (Fig 4C). Next, samples were profiled on Q10 with 100 mM Tris-HCl of varying pH range (pH 6-9) with 0.5% Triton X-100. The optimal condition was pH 8 (data not shown). Subsequently, samples were assessed at pH 8 on Q10 chip, which allows selective binding of compatible proteins with an isoelectric point of 7 or below. Therefore, measurements were repeated on Q10 with samples of 4 independent cultures of WA17 and A9 cells used for profile analysis with 2 μg/spot at pH 8. Plots of PCA scores, based on SELDI spectra, showed a clear differentiation between the A9 and WA17 cell lines (Fig 5A). Moreover, heat map visualisation of A9 and WA17 cells revealed a clear separation by unsupervised hierarchical clustering of the two major subgroups: DS-model (WA17) and normal control (A9) (Fig 5B). These results demonstrate that there is a clear global alteration of protein expression pattern between A9 and WA17 cells.

**Figure 6:**
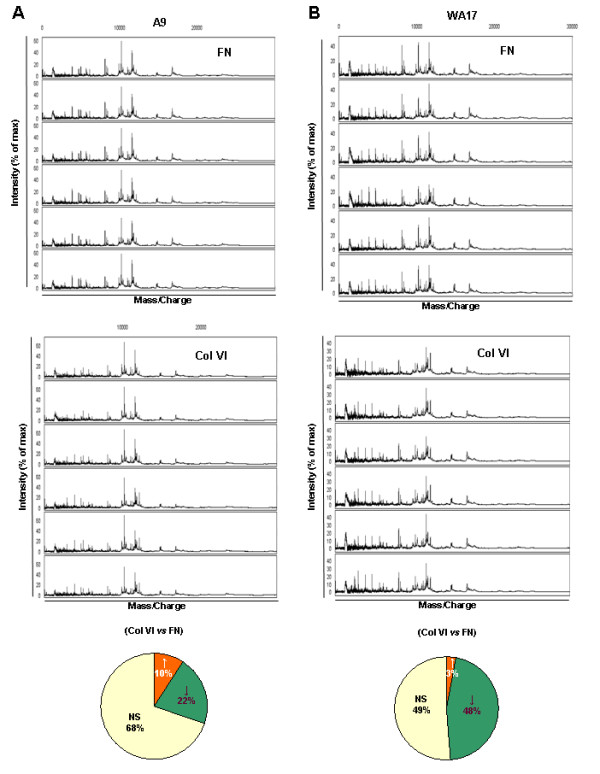
**SELDI-MS analysis of A9 and WA17 cells grown on FN or Col VI**. Cells were grown in presence of FN (5 μg/ml) or Col VI (10 μg/ml). Cells were then lysed and protein profiles were generated by SELDI-TOF MS using the Q10 array. (A) Spectral view for A9 cells in presence of FN or Col VI. Diagram summarizes the response of A9 to Col VI *versus *FN. (B) Same experiments were repeated for WA17 cell line. Significant differences in peak intensity up- (↑) and down- (↓) regulated in ColVI vs FN comparison and "NS" for no significant difference.

Proteins lysates from A9 and WA17 cells cultured in the presence of FN or Col VI for 72 h (n = 7 for each condition) were allowed to bind to the Q10 surface (binding in 0.1 M phosphate buffer, pH 8 supplemented with 0.1% triton X-100), and analysed by SELDI-TOF. A total of 118 peaks were detected on the Q10 surface, with 96 peaks in the range if 2-30 kDa, when peak definition criteria were set to S/N-ratio of 3 or more and peak threshold set to 25% of spectra. From this group, 31 peaks were significantly differentially expressed (p < 0.05; Table 1). For these peaks the intensity levels were compared for each of the two culture conditions to search for differentially represented proteins, or compared for cell type (Table 1). The comparison Col VI *versus *FN, in the Fig 6A and 6B, showed WA17 cells had more ECM-regulated proteins (~50%, diagram right) than A9 cells (~30%, diagram left). This result is the first demonstration that the inside-out signaling is at least as important as the outside-in signaling in explaining the aberrant response of DS cells to the ECM composition.

**Table 1:** Cell proteins found on Q10 surface using SELDI-TOF MS.

	COL6 vs. FN		WA17 vs. A9	
		
Protein mass (Da)	**A9**	**WA17**	**Col6**	**FN**
2928.711474	↑	ns	↑	↑
4703.857024	ns	ns	↑	ns
4981.209087	ns	↓	↓	↓
5679.754004	ns	ns	↓	ns
6114.720974	↓	ns	ns	ns
8103.058978	↓	↓	ns	ns
8312.547561	↓	↓	ns	ns
8441.626517	↓	↓	ns	ns
9903.636707	ns	↓	↓	↓
10213.32293	ns	↓	↓	↓
10422.68469	ns	↓	↓	↓
10469.92325	ns	ns	ns	↑
11029.05511	ns	ns	↓	↓
11213.49186	↑	↓	ns	↑
11592.0861	ns	ns	↓	ns
11787.84126	ns	↑	ns	ns
14764.39772	ns	↓	↑	↑
14868.10077	ns	↓	ns	↑
16795.05992	↓	↓	ns	ns
16975.61298	↓	↓	↑	ns
17144.40172	↓	↓	↑	ns
17710.57534	↓	ns	↑	↑
19940.69626	ns	↓	↓	↓
20990.77856	↑	ns	↓	ns
23283.49199	ns	ns	↓	↓
29302.71775	ns	↓	ns	↑
32146.69417	↓	ns	ns	ns
34916.22347	ns	ns	↓	↓
57962.25434	ns	ns	↑	ns
66566.11805	ns	↑	↑	↑
80036.67172	ns	ns	↑	ns

Significant (p < 0.05, ANOVA with Fisher's *post hoc *test) up (↑) and down (↓) peak intensity differences in Col VI compared to FN are noted. The "ns" denotes no significant differences.

### Proteomic versus phenotypic profiles of A9 and WA17 cells

**Figure 7:**
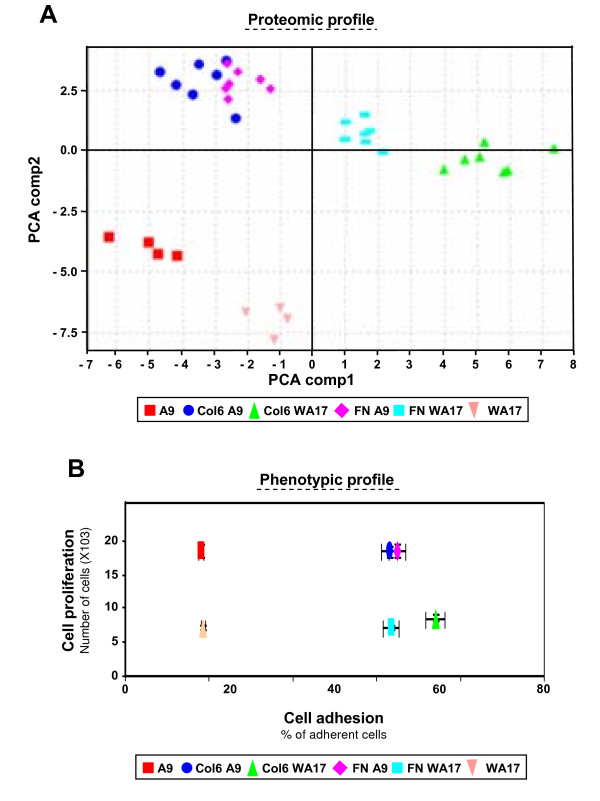
**Proteomic and Phenotypic profiles of A9 and WA17 cell lines**. (A) WA17 and A9 cell lines were grown in presence (n = 14 experiments for each cell line) or absence (n = 4 experiments for each cell line) of Col VI or FN and protein profiles were generated by SELDI-TOF MS using the Q10 array. A correlation biplot of PCA components 1 and 2 is shown (CiphergenExpress software). (B) Plates were coated or not coated with FN or Col VI. Cell adhesion was analyzed on the X axis (n = 12 experiments for each data point) and cell proliferation on the Y axis (n = 12 experiments for each data point). Adhesion and proliferation assays were performed in the same conditions as for the data in figure 3.

We have investigated proteomic profiles in 4 independent cell cultures of A9 and WA17 in absence of ECM (Fig 5), and in 7 independent cell cultures of A9 and WA17 in presence of Col VI or FN (Table 1 and Fig 6). In order to visualize the effects of the response of A9 and WA17 cells cultured in presence or absence of Col VI or FN, a principal component analysis (PCA) was performed on the pattern of intensities of the 31 significant peaks (Fig 7A). Two important conclusions can be drawn from the results of such analysis. Firstly, samples from cells cultured in presence of Col VI and FN clustered differently from cells cultured in absence of ECM, verifying that cellular proteome response to ECM is reproducibly detected by the SELDI-TOF MS analysis. Secondly, proteomic profiles from 14 independent WA17 samples separated into two distinct clusters due to differences in ECM-type (FN or Col IV) while the 14 independent A9 samples projected into a single cluster (Fig 7A). Thus, for the proteomic profiles, we observed clear clustering driven by the presence of the supernumerary HSA21 and correlating with the cell adhesion properties of the two cell lines.

Next, we examined the phenotypic profiles (cell proliferation and cell adhesion), to visualize the effects of the response of A9 and WA17 cells cultured in presence or absence of Col VI or FN, on the cell proliferation and cell adhesion (Fig 7B). For the A9 cells, those cultured in the presence of FN co-clustered with those cultured in the presence of Col VI (Fig 7B). Interestingly, for WA17 cells, those cultured on FN clustered separately from those cultured on ColVI (Fig 7B). Thus, we observed a very good correlation between the cell adhesion/growth properties and their proteomic profile clustering.

## Discussion

Hypothetical paradigms for the molecular origins of DS phenotypes include the gene non-specific developmental instability model and the specific HSA21 gene overdose model [2,24,25]. Several single HSA21 gene overdose models have been linked to phenotypic traits of DS in transgenic mice [26-29] (SOD, ets2, SIM2, DYRK1A), but these models examine a single gene-overdose at a time, without the context of the trisomy of all other genes on the chromosome. Transchromosomic models of DS [18,30,31], constructed by introducing a supernumerary HSA21 into a normal mouse cell, have produced a first mouse with a human chromosome, the most complete HSA21 trisomy model, displaying a range of DS-like phenotypes, including the congenital AVC defect [32]. Transchromosomic models of DS offer a further advantage of the possibility of specific transcriptional reduction-to-disomy of a single gene from the supernumerary human chromosome while maintaining the trisomic expression of all other HSA21 genes [17].

In this study, we establish within a pair of cell lines of a transchromosomic model of DS the two main cellular phenotypes (i.e. decreased cell migration, increased adhesion to Col VI) relevant to the current hypothetical model for the pathogenesis of AVC in DS. A stochastic model [6] had been previously proposed to explain how alterations in the properties of developing endocardial cells could control the formation of endocardial cushions in normal persons, in subjects either from families with a predisposition to congenital heart defects, or with trisomy 21. Normal and abnormal outgrowth of the endocardial cushions of the atrioventricular (AV) canal were modeled by computer simulations. Computer simulations illustrated how decreased cellular migration and increased cellular adhesiveness of fibroblasts from the endocardial cushions of the AV canal in Down syndrome may result in AV canal defects [6]. It has been demonstrated that skin fibroblasts explanted from DS individuals displayed increased adhesion on Col VI [9], a component of the ECM expressed in the developing AVC [11,12]. By examining cell adhesion to purified extracellular matrix components following a 1-hour incubation period, the current study compared the adhesion properties of WA17 (transchromosomic) and A9 (non-trisomic fibroblasts) cell lines (Fig 3A). Cell lines demonstrated similar adhesion profiles on FN, but WA17 cells displayed increased adhesive capacity for Col VI compared to A9 cells (Fig 3A). Although WA17 fibroblasts are not equivalent to embryonic AV canal tissues [33], they represent a reasonable alternative to begin comparing molecular and cellular processes in DS and non-DS tissues. Indeed, our results reproduced the adhesion properties of skin fibroblasts from DS subjects [9]. In addition, we establish, for the first time in a DS model, a phenotype of defective cell migration (which was predicted by a computer simulation for the stochastic Kurnitt model, [6]). Surprisingly, we show that this migration defect does not seem to be caused by the increased adhesion of the cells; on uncoated culture dish surface (where our scratch assay found decreased migration of DS-model cells, Fig 2A and 2B), the WA17 cells adhered as efficiently as the A9 cells (data not shown). This is a new insight, and it allows to speculate that perhaps the two defects are independent of each other, hence might be caused by different mechanisms, and that different HSA21 genes (or groups of genes) in trisomy might be causing decreased migration, whereas other genes may be causing the increased adhesion to ColVI, but both (sets of) genes might be required for the AVC defect to develop. This hypothesis gives even more importance to modeling both phenotypes in a transchromosomic system, where human-specific gene silencing could correct individual gene(s) expression to disomy, while maintaining the trisomic status of the rest of HSA21 [17], and thus help dissect the individual gene(s) contributions to each of the two phenotypes.

Several studies have shown a diminished rate of cellular proliferation in fibroblasts from Down syndrome compared to normal donors [22,34,35]. In Fig 3B, we showed that this defective proliferation is maintained in the transchromosomic fibroblast cell line WA17, irrespective of the adhesion matrix (FN or ColVI). Interestingly, we show that adhesion to Col VI stimulated the proliferation of WA17 cells. To our knowledge, it had never been reported that the adhesion onto a Col VI matrix increased the proliferation of DS cells, and this effect could be another factor to consider occurring during the abnormal development of the AV canal in DS.

The SELDI-TOF peak profile of the normal cells (A9) showed a 30% difference when grown on ColVI compared to FN as the adhesion matrix. This shows that the composition of the ECM, especially in DS where the ECM content of ColVI is higher, may influence the protein expression ("outside-in" signaling). However the comparative data on the DS-model cells (WA17) show that a much larger upheaval of the proteome (close to 50%) is influenced by the adhesion matrix type. This demonstrates that the "inside-out" signaling is equally significant, i.e. the specific response to the change in the ECM composition is heavily affected by the increased dose of HSA21 genes. This suggests that the function of one or more HSA21 proteins is linked to the adhesion properties and the cellular response to the ECM stimuli. This is potentially a very important new insight, because the cellular response to changes in extracellular matrix can also trigger epithelial-mesenchymal transformation (EMT) [7], the developmental process thought to be responsible for heart valve and septa development [8]. The list of peaks showing a change in intensity or presence (Table 1) when ColVI is introduced instead of FN, can be reproducibly modeled in this system (Fig 6 and 7), offering the possibility to dissect the contributions of individual HSA21 genes within the trisomy to the specific proteomic changes. More in-depth studies are needed to identify the proteins behind these peaks, using electrospray nano-LC-MS/MS and related approaches. The overdosed HSA21 genes causing the adhesion and proliferation differences may be similar or same to the ones causing the proteome perturbation, as suggested by the data in Fig 7, where differing proteomic peaks of the DS-model cells (unlike those of normal cells) split into two clusters, one grown on FN and one grown on ColVI, and so did their phenotypic profile, based on adhesion plotted against the proliferation.

## Conclusion

In this study, we show that increased DS cell adhesion to ColVI as matrix, aberrant proliferation of adhering DS cells, and aberrant cell migration (independent of adhesion) can all be reproduced in a transchromosomic model of DS (WA17) [14]. Transchromosomic models of DS offer a further advantage of the possibility of specific transcriptional silencing of a single gene from the supernumerary human chromosome while maintaining the trisomic expression of all other HSA21 genes [17], thereby assigning the causative genetic contribution for a phenotype to the trisomic overdose of a single HSA21 gene [17]. We show that trisomy 21 cells, to a much greater degree than normal controls, acquire SELDI-TOF-MS detectable proteome changes specific to the presence of collagen VI as adhesion matrix. Our data provide an indication, at the proteomic level, that trisomy 21 affects the cell-autonomous proteome response to the change in the extracellular matrix composition.

In conclusion, this set of experiments establishes a cellular model system capable of dissecting the specific HSA21 gene-overdose contributions to aberrant cell migration, adhesion, and the proteome response to collagen VI, which could further inform the attempts to identify and model the genetic cause of the pathogenesis of CHD in DS.

## Methods

### Materials and Cell culture

Purified bovine Collagen VI (COL VI) and human fibronectin (FN) were from Chemicon (Temecula, CA). Bovine serum albumin (BSA) and other general reagents and tissue culture media in this study were from Sigma (Dorset, UK) unless otherwise stated. A9 and WA17 cells [18], a kind gift of Prof. E.M. Fisher, were passed every three days at 3 × 10^3 ^cells/cm^2 ^in Dulbecco's Modified Eagles Medium (DMEM) supplemented with 10% fetal bovine serum and maintained in an atmosphere of 5% CO2 humidified air at 37°C.

### PCR analysis

Analysis of the presence of HSA21 in WA17 was carried out using human-specific primer sequences: USP25-F AGTAGCGAAACAGTGCATTAC, USP25-R GATGAGGTCACACCTGAATAG, MIR155b-F TACTATATGCTGTCACTCCAG, MIR155b-R AGGTTGAACATCCCAGTGAC, RUNX-F AAGATGAAACGTGGAGA AATAG, RUNX-R CTGGACATCACCCACGAGTG, D21S212-F CATTTTAATGAACACCGCTC, D21S212-R GGCCTCCTGGAATAATTCTC. Mouse keratin19 primers were used as positive control: Krt19-F AACCGGAAGGATGCTGAAG, Krt19-R GACTGCAGCTCAATCTCAAG. Programme 95°C 1 m 30 s, (95°C 30 s, 57°C 30 s, 72°C 30 s) 35 cycles, 72°C 9 m 30 s. For D21S212, the annealing temperature was 53°C.

### Ultra-high resolution array CGH

For detailed structural HSA 21 aberration detection, high resolution NimbleGen HG18 chromosome 21 specific 385K arrays were used, (B3752001-00-01; Roche NimbleGen Systems, Madison, Wisconsin, USA). The 385K average probe distance was 70 bp. DNA labeling, array hybridization, post-hybridization washes and scanning were essentially performed according to the manufacturer's instructions (Roche NimbleGen, Madison, Wisconsin, USA). The acquired images were analyzed using NimbleScan V2.4 extraction software (Roche NimbleGen, Wisconsin, USA). For each spot on the array, log2 Cy3/Cy5 ratio, was calculated using the segMNT algorithm, which also applied an automatic segment detection. The relative intensity of the test sample (Cy3 labeled WA17) vs. the reference DNA (Cy5 labeled A9 DNA) indicated on a log2 scale. A positive result was determined when a genomic segment complementary to oligonucleotide probes for CNV (gain or loss) was 0.2 fold average difference from reference normal DNA. A 50× averaging window was also calculated, resulting in 3500 bp segments for this array. Based on previous experiments (data not shown), the few p-arm probes, as well as sub-centromeric probes until genomic position 14.4 Mb were not considered in the analyses, due to repeat content (e.g. segmental duplications). The lack of copy number rearrangements of the mouse genome between the WA17 and their parental control A9 cell lines was verified using MM8 WG CGH Whole Genome Tiling Arrays. Data were visualized in SignalMap V1.9 software (Roche NimbleGen, Wisconsin, USA). Averaging windows were used for breakpoint determination.

### Cell Migration, Adhesion and Proliferation Assays

The *in vitro *scratch assay was performed using A9 and WA17 cell lines to measure cell migration as previously described [19]. A9 and WA17 cells were grown until confluent, and treated with 10 mg/ml mitomycin C for 2 h, before a scratch wound was introduced using a yellow pipette tip. Cells were then incubated for 3 days in normal growth media. Pictures were taken at 0, 1, 2 and 3 days after wounding. Four non-overlapping scratch wounds of each of 3 independent cultures (n = 12 data points for each cell line) were microphotographed and cells migrated into the wound site were quantified. Microphotographs were captured by Eclipse TE2000-S microscope from Nikon and cells migrated into the wound site were counted and presented as mean ± SD. Cell adhesion assays were performed in absence or presence of Col VI and FN (n = 12 independent cultures for each cell line and each ECM condition) as previously described [9]. For cell proliferation, cells were plated in 96-well dishes coated or uncoated with purified extracellular matrix components (ECM) ColVI or FN (n = 12 independent cultures for each cell line and each ECM condition). Cell proliferation was measured using a cell counter (NucleoCounter - Chemometec).

### SDS-PAGE and Immunoblot

Cells were solubilized for 30 min on ice in buffer A (30 mM Tris-HCl pH 8.0, 150 mM NaCl, 1 mM PMSF, 1 mM NaF, 1 μg/ml leupeptin, 5 KU/ml aprotinin containing 1.5% Triton-X100). The lysate was clarified by centrifugation at 435,000 × *g*_*max *_for 30 min at 4°C. SDS-PAGE and immunoblot analyses were performed as described previously [20].

### Protein profiling by SELDI-MS TOF

After 3 days of culture in presence or absence of ECM, harvested cell pellets of A9 and WA17 lines were resuspended in a lysis buffer (50 mM Tris-HCl (pH7), 150 mM NaCl, and 1% Triton-X100). Whole cell lysates were centrifuged at 435,000 × *g*_*max *_for 30 min at 4°C. Total protein concentration was determined by Bradford assay. Cell extracts were diluted in the appropriate binding buffers for the different chromatographic Protein Chip arrays. Four arrays types, each with a different binding condition, were used for profiling the cell lysates. The following binding buffers were used for washing and protein binding on the arrays: weak cationic exchange (CM10) with 100 mM NaOAc4, 0.1% Triton X-100; hydrophobic array (H50) with 10% ACN, 0.1% TFA; strong anion exchange array (Q10) with 50 mM Tris, pH 9.0, 0.1% Triton X-100; and the IMAC Ni array was activated with 100 mM NiSO4 for 15 min and used with PBS buffer, 0.1% Triton X-100. Arrays with sample and peptide/protein molecular weight standards (Bio-Rad) were analyzed using a PBS IIc ProteinChip Reader system (Ciphergen Biosystems). For data acquisition, a mass range of 2 to 150 kDa was captured, with optimisation range set from 2 to 30 kDa. Spectra were normalized to TIC and analyzed by the ProteinChip software and CiphergenExpress software.

Spectra were clustered in CiphergenExpress using the following settings: first pass 5.0 S/N (signal to noise) 3.0 valley depth; minimal peak threshold: 25% of all spectra.; second pass: 3.0 S/N; 3.0 valley depth. The software was used to detect peak differences by the Mann-Whitney U test for nonparametric data sets and the Kruscal-Wallis test. All spectra were inspected manually to validate peak differences. *p*-values were calculated using the Mann-Whitney *U *test for non-parametric data sets. The calculation of intensity ratios and comparison of the groups as well as hierarchical clustering and heat map analysis were performed using tools integrated in the CiphergenExpress software.

### Statistical analysis

Statistical computation and estimation of significance were carried out using unpaired Student's *T*-test using Prism4 (GraphPad Software). The computed 2-tailed *T*-test p values were considered highly statiscally significant when p ranges from 0,001 to 0,01 (noted as ** in Fig 2B and 3A), significant when 0,05 > p > 0,01 (noted as * in Fig 3B), and not siginificant when p > 0,05. Differences in peak intensity were evaluated using ANOVA with Fischer's post hoc test (SELDI-TOF MS, multigroup comparisons). *P*-values lower than 0.05 were considered significant.

## Competing interests

The authors declare that they have no competing interests.

## Authors' contributions

FD designed the experiments and carried out the majority of experiments conducted in this study, and wrote the manuscript. EB was responsible for generating the raw data and primary analysis of the SELDI-TOF experiments. AH was responsible for generating the raw data and primary analysis of the high resolution array experiments. JV was responsible for generating the final interpretation of the high resolution array experiments. JG helped with secondary proteomics data analysis and interpretation. FEC organised, established and supervised the use of SELDI-TOF technology in our institute. DN was scientific lead and responsible for the supervision and writing of the manuscript. All authors read and approved the final manuscript.
